# The approximate entropy of the electromyographic signals of tremor correlates with the osmotic fragility of human erythrocytes

**DOI:** 10.1186/1475-925X-9-29

**Published:** 2010-06-22

**Authors:** Paulo HG Mansur, Lacordaire KP Cury, José OB Leite, Adriano A Pereira, Nilson Penha-Silva, Adriano O Andrade

**Affiliations:** 1 Biomedical Engineering Laboratory, Faculty of Electrical Engineering, Federal University of Uberlândia, Uberlândia, Minas Gerais, 38.408-100, Brazil; 2 Institute of Genetics and Biochemistry, Federal University of Uberlândia, Uberlândia, MG, 38400-902, Brazil

## Abstract

**Background:**

The main problem of tremor is the damage caused to the quality of the life of patients, especially those at more advanced ages. There is not a consensus yet about the origins of this disorder, but it can be examined in the correlations between the biological signs of aging and the tremor characteristics.

**Methods:**

This work sought correlations between the osmotic fragility of erythrocytes and features extracted from electromyographic (EMG) activity resulting from physiological tremor in healthy patients (N = 44) at different ages (24-87 years). The osmotic fragility was spectrophotometrically evaluated by the dependence of hemolysis, provided by the absorbance in 540 nm (*A*_*54*_*o)*, on the concentration of NaCl. The data were adjusted to curves of sigmoidal regression and characterized by the half transition point (*H*_*50*_), amplitude of lysis transition (*dx*) and values of *A*_*540 *_in the curve regions that characterize the presence of lysed (*A*_*1*_) and preserved erythrocytes (*A*_*2*_). The approximate entropy was estimated from EMG signals detected from the extensor carpi ulnaris muscle during the movement of the hand of subjects holding up a laser pen towards an Archimedes spiral, fixed in a whiteboard. The evaluations were carried out with the laser pen at rest, at the center of the spiral, and in movement from the center to the outside and from outside to the center. The correlations among the parameters of osmotic fragility, tremor and age were tested.

**Results:**

Negative correlations with age were found for *A*_*1 *_and *dx*. With the hand at rest, a positive correlation with *H*_*50 *_was found for the approximate entropy. Negative correlations with *H*_*50 *_were found for the entropy with the hand in movement, as from the center to the outside or from the outside to the center of the spiral.

**Conclusion:**

In healthy individuals, the increase in the erythrocyte osmotic fragility was associated with a decrease in the approximate entropy for rest tremor and with an increase of the entropy for movement tremor. This suggests that the neuromuscular degeneration associated with tremor entails also the mechanisms involved in the breakdown of structural homeostasis of the erythrocyte membrane.

## Background

Tremor, the most common movement disorder, is defined as a rhythmic and involuntary oscillation of one part of the body, caused by reciprocally innervated antagonist muscles, which leads to repetitive contractions [1-4]. It may vary in terms of frequency and amplitude and it is influenced by physiological characteristics and the consumption of some types of drugs [2]. There are more than 10 types of tremor, with variations of degrees and progression levels [1].

The main problem related to tremor is the damage caused to the patients' life quality, especially those at more advanced ages. It can lead to physical and social deteriorations and constraints in the manipulation of objects [2]. The majority of persons consider that tremor is a consequence of normal aging; thus, it is not described in medical records, and therefore, patients may not receive adequate treatment [3].

Essential tremor and Parkinson's disease are the main pathological causes of tremor in elderly people. Their etiologies have still not been totally clarified. There is evidence for the involvement of oscillation generators in the central nervous system. The most important of these are the bulbar olive, the red nucleus and the ventromedial nucleus of the thalamus, which together constitute the olivary-cerebellar-thalamic circuit. Besides, there is the oscillatory activity generated by the basal ganglia, which is the more affected region in Parkinson's disease. Since such structures are connected, it is not possible to establish exactly which one is the responsible for the diagnosed tremor in each patient [5].

Due to these reasons, studies of the tremor have received the attention of many research centers all over the world, with the aim to obtain a better understanding of the relations between this disorder and the characteristics that may clarify its origins or facilitate the early diagnosis [6].

Tremor measurements are accomplished with equipment that collects the neuro-electric signals that generate tremor, such as the electromyographic [7-9] or electroencephalographic [7,10,11], or that quantifies directly these oscillations, such as the accelerometer [12-14] or the spirograph [15-17]. These measurements do not present additional difficulties, since the available instruments are widely known and used.

Although the literature on tremor is complex, it shows that it is possible to differentiate the pathological from the non-pathological tremor, and understand their origins and to establish reasonable strategies for monitoring and treatment of the patient [1,2,18,19].

However, there is no consensus regarding the origin of the physiological tremor. It has been attributed to increase in the destruction of dopamine-producing cells that occurs progressively with increasing age [20-25]. Such destruction would be the cause of the non-Parkinson's tremor associated to aging.

This degeneration could be just 'regional' and restricted to certain nerve cells or it could manifest itself widely throughout all the body cells. A widespread or 'universal' degeneration could be due to changes in the conditions governing the homeostasis of biological structures. This type of degeneration would have cumulative and irreversible implications. Its consequences would be more serious for post-mitotic cells that are non-renewable or slowly renewable cells, such as neural cells, but much less serious for renewable post-mitotic cells, such as erythrocytes.

The physiologic tremor could be a 'regional' manifestation of a 'universal' degeneration process such as a structural homeostasis break in the cell membranes.

In this study, this hypothesis is tested using the red blood cells (RBC) or erythrocytes as a model for study of the cell membranes. The composition and behavior of erythrocytes shall reflect the homeostatic chronic conditions common to other post-mitotic cells of the body. In fact, the erythrocytes have been the most commonly used cells to study the effects of degeneration on the body [26-30].

The test of such hypothesis is based on assessment of the correlation between the electromyographic variables associated with the physiological tremor and the stability of erythrocyte membrane in healthy subjects.

## Methods

### Subjects

The work was previously approved by an institutional Ethics Committee. The volunteers (N = 44; 24-87 years) were recruited among the students of the Faculty of Physical Education and patients of the Clinical Hospital of the Federal University of Uberlândia. None of them had a history or any clinical evidence of neuromuscular disorder, duly certified by a neurologist. An Informed Consent Term was obtained from each volunteer who agreed to participate in the study.

### Evaluation of the Osmotic Fragility of Erythrocytes

Aliquots (1 mL) of solutions containing NaCl at 0.1; 0.2; 0.3; 0.4; 0.42; 0.43; 0.44; 0.46; 0.48; 0.50; 0.53; 0.56; 0.6; 0.7; 0.8; 0.9 and 1.0 g.dL^-1 ^were added to Eppendorf tubes. After pre-incubation in a water bath at 37°C for 10 minutes, the tubes received aliquots of 10 μL of blood and then they were hermetically sealed, homogenized and incubated in a thermostat controlled bath (37°C) for 30 minutes. After incubation, the tubes were centrifuged at 1300 × g for 10 minutes and their supernatants were removed for measurement of their absorbance values at 540 nm (*A*_*540*_) in a UV-VIS spectrophotometer (Shimadzu, Model UV-1650, Kyoto, Japan). The dependence of *A*_*540 *_on the salt concentration was adjusted to a sigmoidal regression line, according to the Boltzmann equation,

where *A*_*1 *_and *A*_*2 *_are, respectively, the average values of *A*_*540 *_in the first and in the second plateaus of the sigmoid, *x *is the NaCl concentration, *H*_*50 *_is the NaCl concentration in which occurs 50% of hemolysis and *dx *is the concentration range of NaCl in the sigmoidal transition between *A*_*1 *_and *A*_*2*_. The adjustments were accomplished using the software OriginPro 7.5 (Microcal, Northampton, Massachusetts, USA).

**Figure 1:**
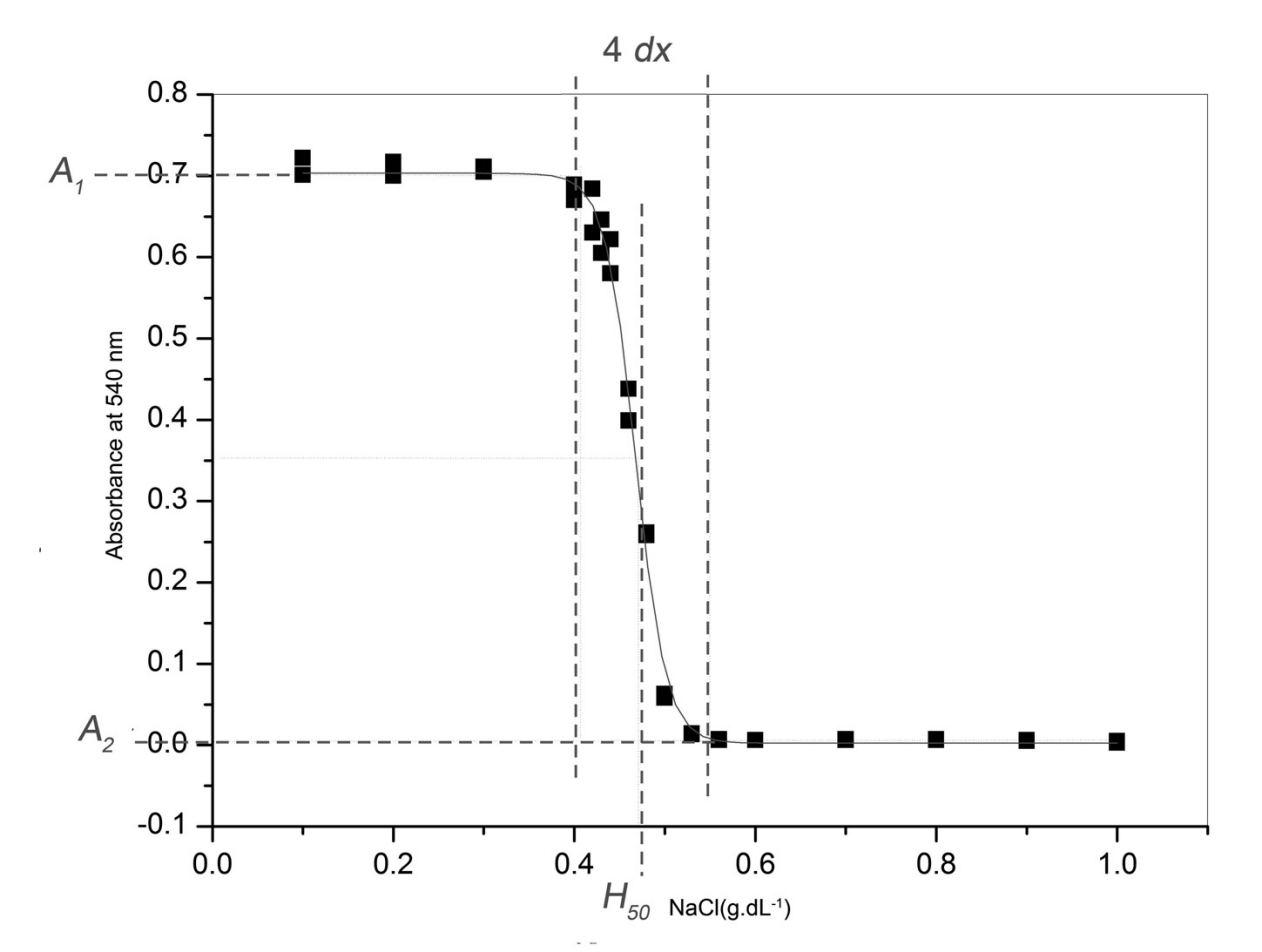
**A typical curve of sigmoidal regression used in the determination of the osmotic fragility of erythrocytes**. The graphical meaning of the parameters evaluated in each situation (*A*_*1*_*, A*_*2*_*, dx *and *H*_*50*_) are shown.

Figure 1 illustrates these parameters. *A*_*1 *_is given by the maximal stationary values of absorbance at 540 nm and is proportional to the amount of hemoglobin released in the blood sample under the extreme hypotonic conditions of the experiment. *A*_*2 *_is given by the minimum stationary values of absorbance at 540 nm and represents the amount of hemoglobin released in the blood sample under isotonic and mild hypotonic conditions. *H*_*50 *_and *dx *are the variables that represent properly the osmotic fragility of the erythrocytes. Complete descriptions of this method and its rationale can be found in literature [31-33].

### Collection of EMG signals and calculation of the approximate entropy

During the evaluation of tremor, each volunteer was kept comfortably seated in an upright chair, with feet flat on the floor, forming angles of 90° between the back and seat and between the seat and legs. The forearm was supported on a pedestal placed in front, so as to form an angle of 90 degrees with the trunk and parallel to the floor. At 80 cm from the pedestal, a spiral of Archimedes was placed on a white screen, so that the center of the spiral was perfectly aligned with the hand of the individual, where a laser pointer was placed. The individual was instructed to remain static (30 seconds) and, following the researcher instructions, move the laser pointer from the outside toward the center and then from the center toward the outside of the spiral, with 2 minutes between each sequence. The procedure was performed with each hand. During the experiment, a surface electrode was placed on the 'extensor carpi ulnaris' muscle for the registration of EMG activity (MyosystemBr1-P84, DataHominis Tecnologia Ltda, Brazil).

In this study, the approximate entropy of the tremor was estimated from EMG signals according to the algorithm described in Pincus [34]. Those signals represent the bioelectrical stimuli sent by brain to activate the motor units of muscles, which are responsible for both normal movements and tremor activities. In Statistics, the approximate entropy is used to establish the uncertainty or variability of a system. Thus, the approximate entropy calculated from the EMG signal, is dependent on the variability of its amplitude and frequency. It represents better the variability of the EMG signal than only the amplitude or only the signal frequency, since the approximate entropy congregates the variability in both variables of the signal. The approximate entropy shall be more appropriate to estimate the physiological tremor associated with the aging process. In general, the larger the variability of the signal (tremor), the larger is the entropy.

A Matlab routine based on the Pincus's algorithm [34] was employed to calculate the approximate entropy of EMG signals collected from each situation (rest, inward movement and outward movement).

### Analysis of the correlation between the osmotic fragility of erythrocytes and electromyographic signals of tremor

Analyses of correlation were performed between the electromyographic variables of the tremor (amplitude, frequency and entropy) and the variables related to the stability of erythrocyte membrane (*A*_*1*_*, A*_*2*_*, H*_*50 *_and *dx*) or the volunteers' age. These analyses were accomplished using a linear regression. In each analysis the 95% confidence interval (CI) was always indicated by lines above and below the regression line. All analyses were performed with the Matlab Statistical toolbox [35]. The values of SSE (Sum of Squares of Error), MSE (Mean Squared Error) or variance, RMSE (Root Mean Squared Error) or standard error, and R^2 ^(Pearson correlation coefficient) were determined in each analysis. The correlations were considered significant when R^2 ^was larger than 0.8. The number of points (N) considered in the analyses was always 44. The occurrence of overlapping of experimental points in some situations can give the visual impression that the value of N is smaller. Attempts to censor possible outlier points in the correlation analysis always returned R^2 ^values above the threshold of significance considered in the work.

## Results

Figure 1 presents typical results obtained in the determination of the stability of the erythrocyte membrane. The experimental points were adjusted to a line of sigmoidal regression, with determination of the parameters *A*_*1*_*, A*_*2*_, *H*_*50 *_and *dx*.

**Figure 2:**
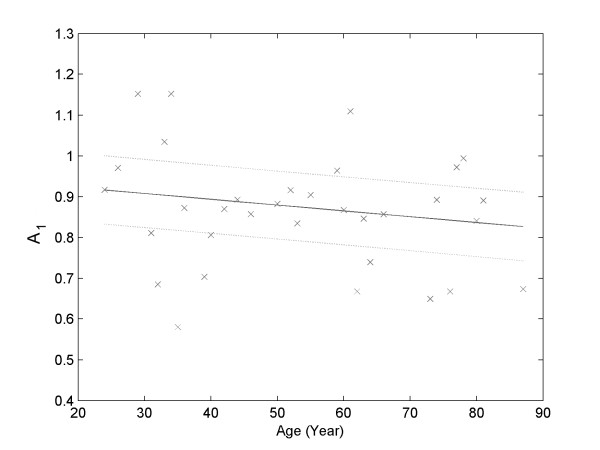
**Linear correlation between *A*_*1 *_and age (*R*^*2 *^= 0.9521)**.

The values of *A*_*1 *_presented a considerably strong and inverse correlation (R^2 ^= 0.9521) with the volunteers' age (Figure 2). Since *A*_*1 *_is the average value of the *A*_*540 *_values in a hypotonic environment where the red cells are completely lysed, *A*_*1 *_expresses the amount of hemoglobin present in the blood of the volunteer. There was a decrease of about 10% in the *A*_*1 *_values of the volunteers in the age range of 80-90 in comparison to that of 20-30 years. This trend agrees with the increase in the prevalence of anemia reported in the most elevated age ranges [36,37].

**Figure 3:**
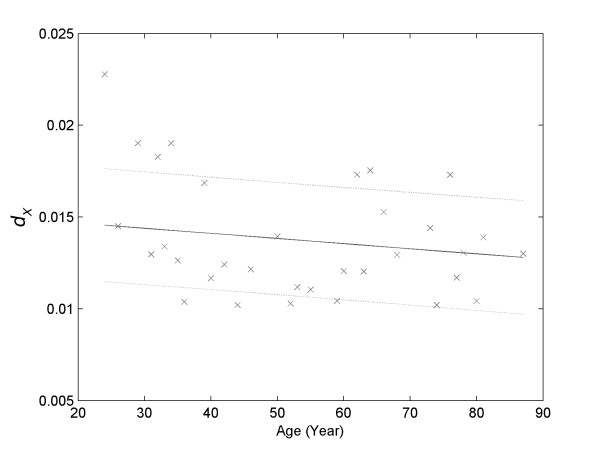
**Linear correlation between *dx *and age (*R*^*2 *^= 0.8800)**.

The *dx *values presented a negative correlation with the volunteers' age (Figure 3). This means that increasing age was associated with the occurrence of hemolysis in narrower ranges of salt concentration. The range of salt concentration associated with hemolysis (*dx*) was 12% lower in the individuals aged 80 to 90 years compared with those from 20 to 30 years.

**Figure 4:**
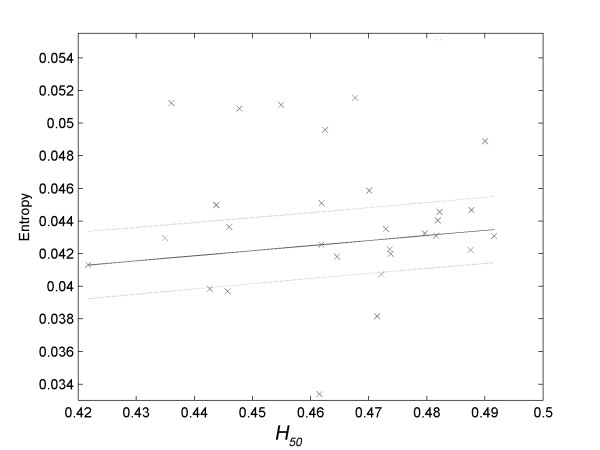
**Linear correlation of *H*_*50 *_with the approximate entropy of the electromyographic data related to the volunteer's hand with the laser pointer at rest at the center of the spiral (*R*^*2 *^= 0.9397)**.

**Figure 5:**
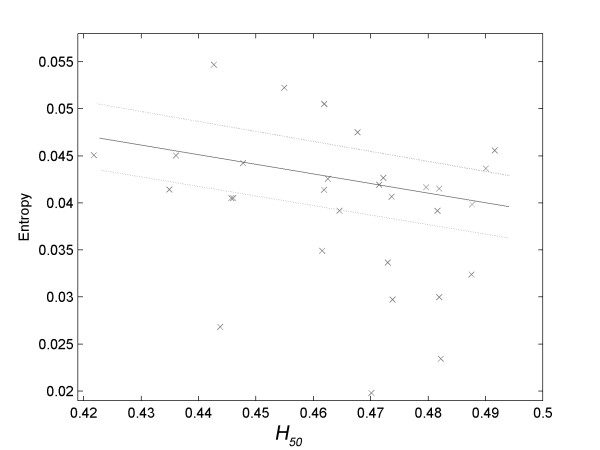
**Linear correlation of *H*_*50 *_with the approximate entropy of the electromyographic data related to the movement of the hand from the interior to the exterior of the spiral (*R*^*2 *^= 0.9374)**.

**Figure 6:**
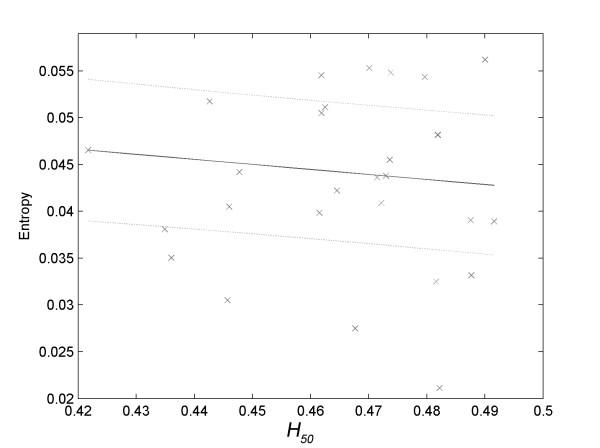
**Linear correlation of *H*_*50 *_with the approximate entropy of the electromyographic data related to the movement of the hand from the exterior to the interior of the spiral (*R*^*2 *^= 0.8350)**.

There was no significance (*R*^*2 *^< 0.800) in the correlations tested between the variables associated to the osmotic fragility of erythrocytes (A_1_, A_2_, H_50 _and dx) and the primary variables of the EMG signal associated with tremor (frequency and amplitude). However, the approximate entropy associated with the electromyographic signals of tremor presented strong correlations with *H*_*50 *_in all situations used for determining tremor, that is, with the hand at rest (Figure 4, *R*^*2 *^= 0.9397) and in movement from outside to the center (Figure 5, *R*^*2 *^= 0.9374) and from the center to outside of the spiral (Figure 6, *R*^*2 *^= 0.8350).

The differentiation of tremor at rest in relation to tremor in movement is important. In the most common diseases, the tremor manifests more at rest (Parkinson's disease) than in movement (essential tremor). Cases where both situations are present at the same intensity are rare [1,4].

With the hand at rest, the approximate entropy of the tremor presented a 'positive' correlation with *H*_*50 *_(Figure 4). This means that individuals with erythrocyte membrane less resistant to hypotonic shock had greater values of approximate entropy of tremor when they kept their hands at rest.

With the hand in movement, the approximate entropy of the tremor presented a 'negative' correlation with *H*_*50 *_(Figures 5 and 6). This means that the individuals with erythrocyte membranes more resistant to hypotonic shock presented greater values of approximate entropy of tremor when they kept their hands in movement.

## Discussion and conclusions

The stability of erythrocyte membrane can be measured in several ways. One of them is by the osmotic fragility against a salt gradient, as done in this work (Figure 1). The osmotic fragility can be evaluated by the half transition point (*H*_*50*_) of the hemolysis curve and salt concentration range (*dx*) necessary to promote the transition between preserved (*A*_*2*_) and lysed erythrocytes (*A*_*1*_).

The *A*_*1 *_parameter describes a behavior of hemoglobin from lysed erythrocytes and is therefore directly linked to the amount of hemoglobin in the blood of the volunteer. The occurrence of a negative linear correlation between the values of *A*_*1 *_and age of the volunteers (Figure 2) should reflect the tendency of older individuals to present lower levels of hemoglobin [37,38].

The greater the values of *H*_*50 *_and the smaller the *dx*, the more fragile are the erythrocytes against hypotonic stress. In this work, the values of *H*_*50 *_showed no correlation with age of the volunteers, but the values of *dx *had a slight negative correlation with age (Figure 3). The existence of this relationship must mean that the age increase would make the red cells more vulnerable to undergo lysis in a narrower range of salt concentration.

The hypothesis that motivated this study is that the physiologic tremor could be a 'regional' manifestation of a 'universal' degeneration process such as a structural homeostasis break that can affect the erythrocyte membrane among other cell membranes in the organism.

If this hypothesis is correct, there should be some correlation between parameters associated with the stability of red blood cells (*A*_*1*_, *A*_*2*_, *H*_*50 *_or *dx*) and variables associated with the electromyographic signal of tremor. In fact, the values of approximate entropy of hand tremor at rest (Figure 4) and in motion (Figure 5 and Figure 6) showed significant correlations with the values of *H*_*50*_.

However, there was an opposite effect for the correlations with the arm at rest with respect to correlations with the arm in motion. The entropy of the rest tremor increased with the decrease in the stability of erythrocytes.

It is possible that this difference is due to the composition of physiological tremor, present in all individuals, even healthy. Part of the signal composing the EMG activity is a result of the mechanical oscillations produced by the cardiac systole, which spreads throughout the body [38-40]. At rest, this component may be more significant than the tremor activity generated by neurogenic signals, a situation that is reversed when muscle cells are activated to perform a movement, which has been observed in other studies [8,39].

However, these results are not consensual, because there are many studies that attribute the origin of physiological tremor to several other factors [40].

The lack of a consensus on this issue encourages the search for explanations based on the composition and behavior of membranes.

All biological membranes of our body have the same kind of structure. They are formed by a double lipid layer where some proteins are peripherally or integrally associated [41]. The nature and function of the membrane proteins are genetically regulated. The formation and insertion of lipids in the membrane is also genetically regulated, but not in an absolute sense [42]. The membrane lipids have compositions that reflect the conditions of fatty acids (by nutrition) in the organism. Humans do not have some genes that express some enzymes (dessaturases) associated to the formation of linoleic and α-linolenic acids, which are important to the biosynthesis of many other polyunsatured fatty acids (PUFA) [43]. This means that the amount of these specific PUFA in the membrane lipids depends on our nutrition. On the other hand, these PUFA are very vulnerable to a phenomenon called lipoperoxidation, which is implicated in many degenerative diseases. Thus, all biological membranes have common components, levels of which depend on nutrition and degenerative mechanisms such as oxidation. In this relative aspect, not in an absolute sense, membranes of erythrocytes may reflect the conditions of other cells in the body. The cholesterol content and the balance between saturated and unsaturated fatty acids in the phospholipids of cell membrane is a critical factor in the determination of the membrane stability [44].

If indeed the stability of the erythrocyte membrane reflects the behavior of cells from brain and muscle, the explanation for that correlation would be the cellular vulnerability to destruction. This makes sense, since an increase in the destruction of dopamine-producing cells occurs progressively with increasing age [20-25]. Such destruction would be the cause of the non-Parkinson's tremor associated to aging.

On the other hand, the approximate entropy of the tremor associated to the arm movement, both from the center outwards (Figure 5) and from outside to the center of the spiral (Figure 6), decreased with the decrease in the stability of erythrocytes. This means that the less resistant are the cell membranes of the erythrocytes, the lower the variability of the tremor, regardless of the direction in which the spiral was followed, i.e., the approximate entropy of the signal is higher the greater the stability of membranes.

The explanation for this fact may be in the physiological implications (functionality) of the increased stability of membranes. Stability is a structural property that is essential for the exercise of the specific functions of the biological complexes. To preserve themselves and function properly, the red cells, as well as any biological complexes (protein, nucleic acid and membrane), have to exhibit stability and functionality. The region of larger stability of an organic complex does not necessarily coincide with the region of greater functionality. This issue was reviewed and analyzed by Fields [45] and by Fonseca et al. [46]. Deficiencies associated with lack or excess of stability are the basis of so-called diseases caused by protein misfolding [47]. The specific causes of such protein misfolding diseases can be inherited but also associated with nutrition and life style. Erythrocyte membranes can also have their composition influenced by inherited factors but also by aging, nutrition and life style [48]. Structure and functionality of erythrocytes can be largely affected by changes in the osmolarity of the medium [31,32]. Structure and functionality of erythrocyte membranes can also be affected by changes in the blood levels of lipids. The blood levels of lipids can influence blood viscosity and the structure of the erythrocyte membrane [49], thus affecting its functionality. An increase in stability at the expense of functionality can impair the mechanisms of gas exchange (in the case of erythrocytes) and the transmission of signals (in the case of neural cells and muscle).

Most studies in the literature deal with clinically diagnosed patients with essential tremor or Parkinson's disease, trying to identify characteristics or correlations between these, using various measuring instruments and statistical analyses. This was not the case for this work, whose most important contribution was the study of physiological tremor in a sample of the Brazilian population, which still lacks this type of analysis, calling attention to the fact that the physiological tremor needs to be more properly understood for better understanding of the pathological tremor. The protocols used in this study were effective to detect some trends and possibilities. There was an effective relationship between the approximate entropy of physiological tremor and changes in the behavior of the erythrocyte membrane. It is possible that this relationship is associated with neurological origin of the tremor. However, the sample size is still relatively small and it would be interesting to maximize it, in order to better characterize that trend.

## Competing interests

The authors declare that they have no competing interests.

## Authors' contributions

All authors read and approved the final manuscript. PHGM: data collection, data analysis, writing and revision of the paper. LKPC: data collection and data analysis. JOBL: determination of osmotic fragility data. AAP: data collection, data analysis and revision of the paper. NPS: supervision of experiments on osmotic stability of erythrocytes, data analysis and revision of the paper. AOA: data collection, data analysis, writing and revision of the paper.
